# Transient Combustion Characteristics of Methane–Hydrogen Mixtures in Porous Media Burner

**DOI:** 10.1021/acsomega.4c01065

**Published:** 2024-04-18

**Authors:** Taiming Huang, Xun Ren, Yiyu Chen, Jingmao Ma, Dingxun Yi, Zhongmin Wan, Bo Yu, Wei Zeng

**Affiliations:** † College of Mechanical Engineering, Hunan Institute of Science and Technology, Yueyang 414000, China; ‡ College of Mechanical Engineering, Beijing Institute of Petrochemical Technology, Beijing 102400, China; § Department of Mechanical Engineering, New York Institute of Technology, New York, New York 11568, United States

## Abstract

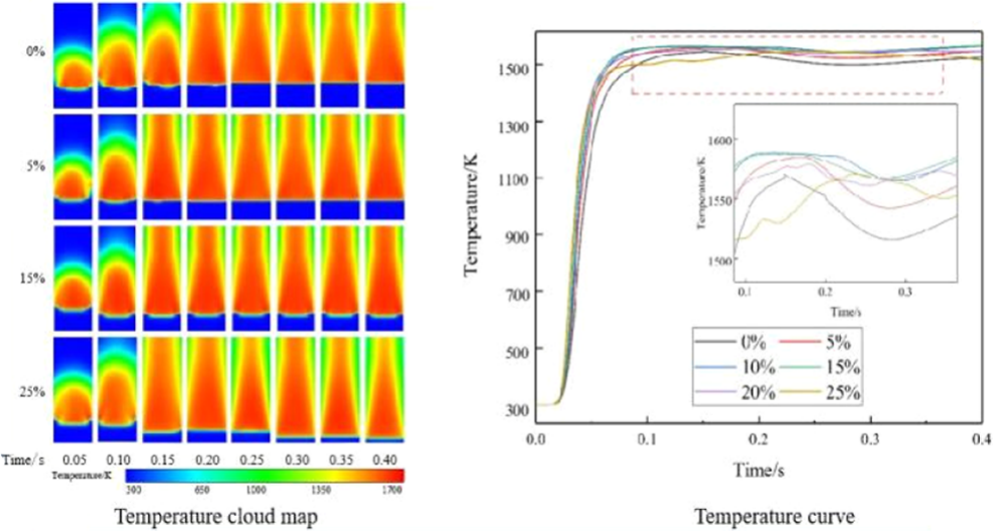

The combustion of conventional methane–hydrogen mixtures is associated with challenges such as combustion instability and excessive pollutant emissions. This study explores the advantages of porous media, which include a wide operating range, enhanced combustion stability, high combustion efficiency, and reduced pollutant emissions. We conducted numerical transient simulations to investigate methane–hydrogen combustion within a porous media, focusing on a cylindrical double-layer porous burner geometry. The research analyzes the temperature, combustion rate, and diffusion characteristics of the methane–hydrogen–precipitated gas flame within the porous media. Additionally, it examines variations in the position and width of the high-temperature region along with changes in carbon and nitrogen emissions. The computations were carried out for different hydrogen blending ratios over the time interval of 0–0.4 s. The results unveil the transient combustion characteristics of hydrogen-enriched methane within a porous media, offering valuable insights for the subsequent optimization of porous media burners (PMB). This study provides a theoretical foundation for enhancing the efficiency and environmental performance of combustion processes involving methane–hydrogen mixtures.

## 1 Introduction

As global energy demands escalate, the use of traditional fossil fuels is putting growing environmental strains. The imperative to enhance combustion efficiency and curtail emissions of combustion pollutants has become a global mandate for sustainable energy production.^1^ In recent years, hydrogen blending combustion technology has emerged as a prominent research focus, owing to its inherent benefits of high combustion efficiency and diminished carbon emissions.^2,3^ This contribution is steering toward a cleaner and more sustainable energy landscape.

Okafor^4^ investigated the influence of hydrogen concentration on hydrogen–methane–air premixed flames. The study results revealed a nonlinear increase in the combustion velocity with increasing hydrogen concentration in the fuel, ranging from 0.00 to 1.00, for all equivalence ratios. Alabas^5^ delved into experimental research exploring the effect of hydrogen enrichment on the characteristics of natural gas turbulent flames. The finding revealed a noteworthy increase in flame speed and combustion rate with rising hydrogen content in methane–hydrogen mixtures. Bhasker’s study^6^ underscored the heightened reaction characteristics of fuel mixtures as hydrogen concentration increased, resulting in shortened combustion duration and reduced total flame length. Investigating the repercussions of hydrogen addition on lean, non-premixed swirling flames of natural gas, Cozzi and Coghe^7^ observed the presence of shorter and narrower blue flames near the head of the burner.

In recent years, porous media premixed combustion has emerged as a dynamic area of research.^8^ This combustion method involves burning fuel–air mixtures within a porous media, presenting advantages such as enhanced flame stability, improved combustion efficiency, and reduced pollutant emissions.^9^ Researchers have extensively explored various facets of this combustion technique to gain a deeper understanding of its fundamental mechanisms and to optimize its performance. Studies have delved into characterizing combustion behavior within porous media, encompassing flame propagation, heat transfer, and pollutant formation.^10^ Experimental investigations, utilizing diverse porous materials and burner configurations, have been conducted to scrutinize their impact on combustion performance.^10,11^ Additionally, numerical simulations have been deployed to unravel the intricate flow and heat transfer phenomena within the porous media.^12^ Furthermore, researchers have delved into the influence of various parameters on porous media premixed combustion, including porous structure properties, fuel–air equivalence ratio, and flow velocity.^13^ These investigations aim to pinpoint optimal operating conditions that maximize combustion efficiency while minimizing pollutant emissions. Moreover, concerted efforts have been made to pioneer advanced diagnostic techniques for the study of porous media premixed combustion, encompassing laser-based techniques, spectroscopy, and imaging methods.^14^ These tools facilitate the measurement and visualization of crucial combustion parameters, such as temperature, species concentrations, and flame characteristics.

In an experimental study, Marbach^15^ investigated the submerged combustion and surface combustion characteristics of CH_4_/air premixed gases within porous media filled with silicon carbide. Their findings indicated that surface combustion exhibited superior combustion limits, while the flame stability of both submerged and surface combustion deteriorated with an increase in the diameter of the silicon carbide particles. Rodenhurst et al.^16^ employed the Damkohler (Da) number to characterize the intensity of surface combustion, revealing a positive correlation between Da and flame intensity. Hashemi^17^ conducted simulation studies on the stable combustion range of CH_4_ premixed gases in submerged combustion under different equivalence ratios (EQRs), observing decreased combustion stability at lower EQR. Investigating the effects of inlet velocity, EQR, extinction coefficient of the porous media, and thermal conductivity on the combustion characteristics of CH_4_ in dual-layer porous media filled with zirconia and alumina, respectively, Liu^18^ provided insights into the intricate factors influencing combustion behavior. Chen^19^ conducted numerical simulations to investigate the effects of hydrogen doping and porous media technology on the combustion characteristics of natural gas. The research findings demonstrate that the use of natural gas with 20% hydrogen doping helps to reduce combustion costs and environmental impacts. A novel two-stage dual-layer PMB was proposed by Jia,^20^ which simulated the combustion performance of the burner under different EQR and different premixed gas inlet velocities. The new burner achieves both complete combustion and energy savings. Liao^21^ proposed the idea of using a PMB to enhance the oxygen–methane combustion reaction rate and expand the stability limits. The combustion characteristics of variable-cross-section (VC) and straight cylinder (SC) structure burners were compared in the simulation study. The results indicate that the flame temperature of VC is lower than that of SC by 200 K, confirming the effectiveness of the VC structure in improving the combustion stability and reducing pollutant emissions. Previous investigations into methane–hydrogen blending within porous media have predominantly concentrated on steady-state combustion with limited exploration of transient combustion. The introduction of hydrogen into methane combustion poses challenges, as it can easily lead to combustion instability. With an escalation in the hydrogen blending ratio, the combustion process becomes progressively intricate, potentially resulting in flame oscillations, flickering, or pronounced combustion instability. Nevertheless, extant studies of steady-state combustion failed to offer a precise explanation for these phenomena. Consequently, this study aims to scrutinize the transient combustion characteristics within a PMB, seeking to provide a comprehensive understanding of these phenomena as a theoretical foundation for the subsequent optimization of PMB. To achieve this, a cylindrical double-layer porous burner geometry was devised, and the transient combustion flame characteristics were examined under the assumption of thermal equilibrium, comparing them with the combustion model proposed by Fursenko’s experiment.^22^

## 2 Model and Numerical Calculation Method

### 2.1 Model and Grid Construction

**Figure 1:**
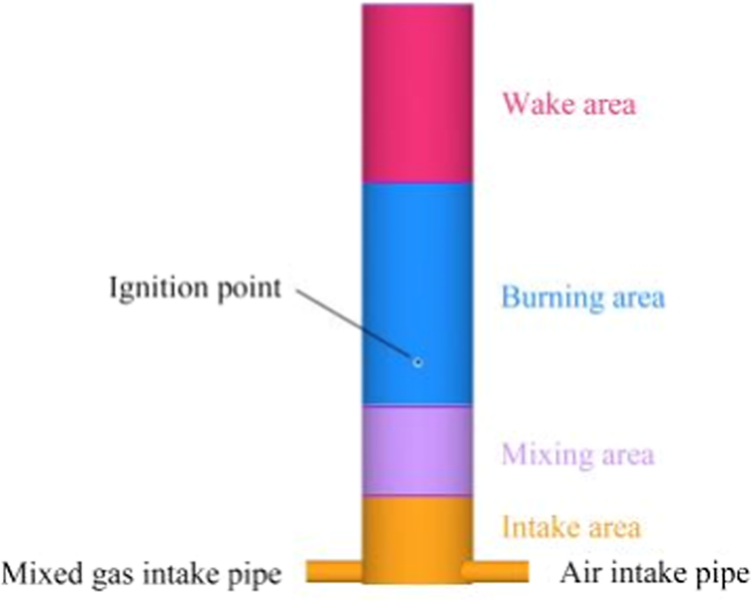
Burner geometry model.

In Figure 1, the geometric model of a dual-layer PMB with multiple cylindrical shapes is illustrated. The model, featuring a diameter of 50 mm and a total length of 260 mm, is segmented into four regions: the intake area, the mixing area, the burning area, and the wake area. This configuration establishes a tangible connection with the actual system, fostering problem-solving based on real-world scenarios and elevating the model’s realism and applicability.

The intake area incorporates two inlet pipes deviating from the central axis, supplying the hydrogen–methane mixture and air. With a diameter of 5 mm, the pipes have an inner diameter of 2 mm for the air inlet and 1.5 mm for the hydrogen inlet. The mixing and burning areas function as porous media zones, while the remaining regions are hollow. The mixing section’s primary role is to facilitate the premixing and preheating of gases, optimizing combustion within the combustion section. The wake section supports gas emission detection and flame observation.

The methane–hydrogen/air premixed gas is introduced into the combustion chamber through the premixing pipeline based on a specified premixing ratio according to the EQR. Ignition occurs at the porous media, forming a stable flame within the porous media region. Combustion byproducts are expelled to the burner’s wake section with the airflow. To ensure ignition and stable combustion, an ignition point is positioned at the burner’s center point, 100 mm away from the burner’s edge, as depicted in Figure 1. The ignition temperature is initially set at 1000K, with an ignition duration is 2 × 10^–3^ s.

**Figure 2:**
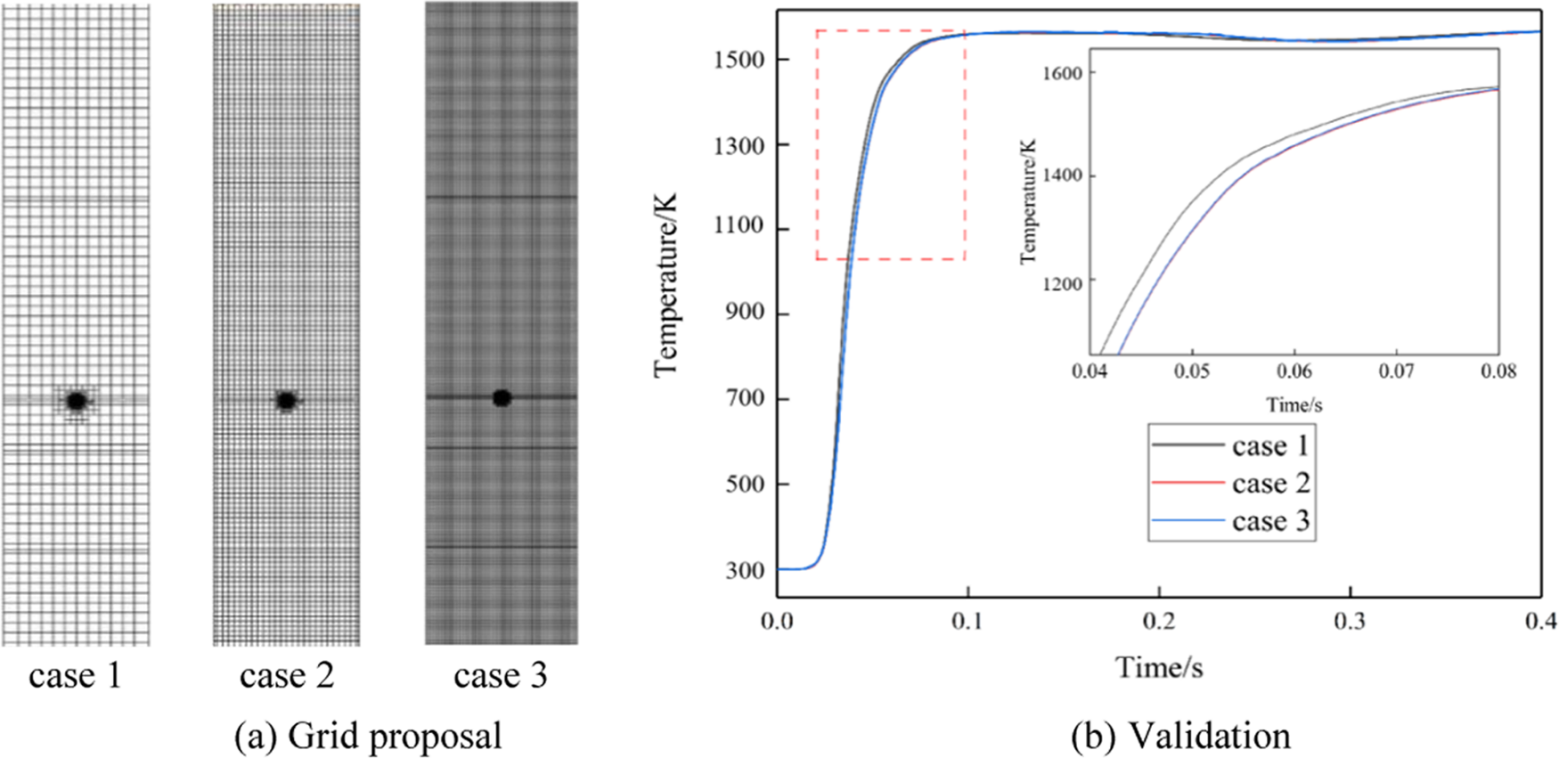
Grid-independent validation.

Figure 2(a) illustrates the computational mesh model of the combustion chamber, where the baseline grid size is set to 4 mm. The computational domain encompasses the intake area, mixing area, burning area, and wake area, with the simulation initiated from the moment of ignition and continued until the flame achieves a stable state.

For grid generation, the Converge software is employed due to the simplicity of the combustion model, offering automatic grid generation and Adaptive Mesh Refinement techniques. This software automatically subdivides the volume grid into orthogonal hexahedral cells, ensuring an efficient and accurate representation of the geometry and flow physics.

In Figure 2(b), grid independence verification is presented, involving various grid densities. This verification specifically focuses on an EQR of 1 with a hydrogen blending ratio of 15%, comparing the average flame temperature at the cross section of the burner at a distance of 130 mm. Table 1 provides the corresponding grid density numbers.

**Table 1:** Grid

proposal	case 1	case 2	case 3
grid density	30,006	177,828	555,582

In case 1, the overall burner configuration remains unaltered with a grid size of 4 mm, as shown in Figure 2(a). Transitioning to case 2, refinement is applied to the walls of the air and premixed gas inlet pipes, as well as other walls, using a 2-level 1-layer mesh (1 mm), while the ignition region undergoes a 4-level refinement (0.25 mm). In case 3, further refinement is introduced, with the walls of the air and premixed gas inlet pipes, as well as other walls, refined using a 3-level 1-layer mesh (0.5 mm), and the ignition region undergoes a 4-level refinement (0.25 mm).

Temperature errors in the case 1 model are notably higher compared with both the case 2 and case 3 grid models, as shown in Figure 2(b). When comparing temperature errors between the case 2 and case 3 models, the difference is a mere 0.12%. Balancing computational efficiency with precision, the case 2 model is chosen as the computational grid. This selection ensures accurate calculations while optimizing computational resources.

### 2.2 Model Selection

In the transient combustion simulations presented in this study, careful selection of turbulence, combustion, emission, and radiation models is pivotal for achieving precise simulation results. Employing the Reynolds-averaged Navier–Stokes (RANS) method^23^ for the turbulence model, the RNG (RNG *k*–ε) method is chosen. This method employs a series of continuous transformations at different spatial scales to offer a coarse-grained description of complex systems or processes, simplifying the problem and facilitating its resolution. Within the RANS method, the RNG *k*–ε model provides predictions closer to experimental measurements than the standard *k*–ε model, particularly in terms of heat transfer, combustion, and pollutant emissions. Consequently, the RNG *k*–ε model is selected as the turbulence model in this study.

The combustion process of methane–hydrogen mixtures is categorized as premixed combustion, where the mixtures and air are premixed before entering the combustion zone. For the combustion model, the SAGE^24^ (Sparse Adaptive Generalized E-Field) chemical solver model and G-Equation combustion model are capable of simulating premixed combustion. In this study, the SAGE chemical solver model is employed, allowing the integration of CHEMKIN input files with detailed chemical kinetic principles for combustion chamber simulations. This involves utilizing a comprehensive chemical mechanism (full mechanism) for the simulations. By combination of SAGE with adaptive meshing, various combustion phenomena such as ignition, premixing, and mixture control can be accurately predicted.

Furthermore, the radiation model^25^ is considered. In the solution of the radiative transfer equation, no additional parameters need to be set, and the material properties can be defined with absorption and scattering coefficients. These thoughtful model selections collectively contribute to a comprehensive and accurate representation of the combustion process for methane/hydrogen mixtures within the PMB.

### 2.3 Model Hypothesis

In the numerical simulation, the following assumptions are introduced:^16^

(1) Porous media does not play a catalytic role in the combustion process.
(2) The methane–hydrogen fuel is thoroughly and uniformly mixed prior to entering the intake manifold.
(3) Both the reactants and products before and after combustion are assumed to be incompressible ideal gases.
(4) The porous media is characterized as isotropic, representing a uniform and dispersed structure.
(5) The impact of gravity on the entire burner is disregarded.
(6) The gas and the solid are in a state of thermal equilibrium

### 2.4 Governing Equation


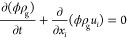
1

where ρ_g_ represents the gas density, *u*_*i*_ denotes the gas velocity vector, and ϕ signifies the porosity.

The momentum conservation equation and turbulent model
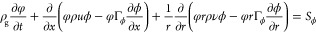
2

where Γ_ϕ_ represents the effective diffusion coefficient, which is defined as
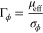
3

The expressions for σ_ϕ_ and *S*_ϕ_ vary in different transport equations, as shown in Table 2

**Table 2:** σ_ϕ_ and *S*_ϕ_ in General Governing Equations

equation	variable	σ_ϕ_	*S*_ϕ_
the *x*-momentum equation	*u*	1	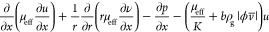 4
the *y*-momentum equation	*v*	1	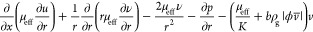 5
the turbulent kinetic energy equation	*k*	1	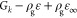 6
the dissipation rate equation	ε	1.3	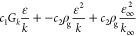 7

Here, *K* represents the permeability, b denotes the inertia coefficient, and μ_*eff*_ signifies the effective viscosity coefficient.

*G*_*k*_ represents the generation term of turbulence, which is given by
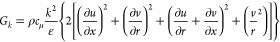
8

where *c*_1_, *c*_2_, and *c*_μ_ are equal to 1.4, 1.92, and 0.09, respectively.

ρ_g_, ε_*∞*_, and  are source terms, where *k*_∞_ and ε_∞_ represent the turbulent kinetic energy and the dissipation rate, respectively.

The energy equation
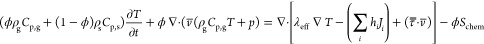
9Here, the subscript g represents gas parameters and the subscript *s* represents solid parameters. *S*_chem_ represents the heat release term caused by chemical reactions, and λ_eff_ denotes the effective thermal conductivity coefficient, defined as follows
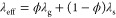
10
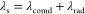
11
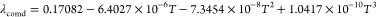
12
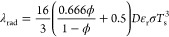
13Here, ε_r_ represents the surface emissivity of the porous media solid and σ denotes the Stefan–Boltzmann constant.

The ideal gas equation
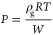
14where *W* represents the average molecular weight of the mixed gas, *R* denotes the universal gas constant, and *P* signifies the pressure.

The equivalence ratio is defined as the ratio of the actual fuel-to-oxygen mixture ratio to the stoichiometric fuel-to-oxygen mixture ratio under chemically equivalent conditions. It is defined as follows
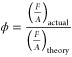
15

### 2.5 Conditions and Parameters

To solve the governing equations, it is imperative to establish appropriate boundary conditions. In this context, the inlet of the combustion chamber is specified as a velocity inlet, while the outlet is defined as a pressure outlet with a reference value corresponding to the standard atmospheric pressure.

**Figure 3:**
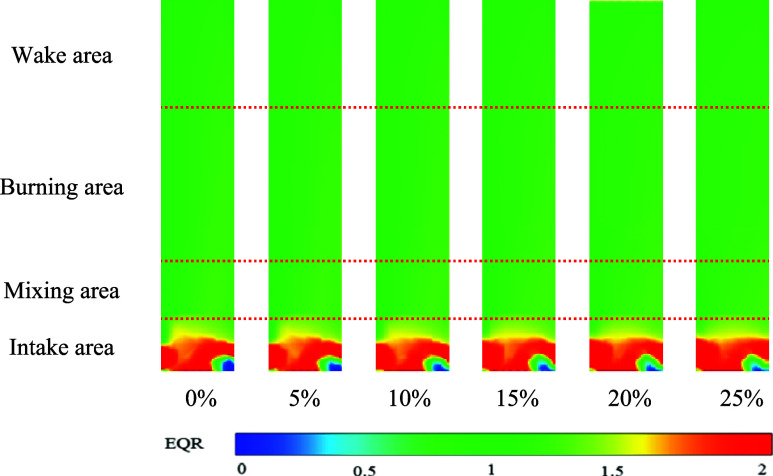
Schematic representation of the equivalent ratio.

Experimental investigations were undertaken to study the combustion characteristics of methane–hydrogen–precipitated gases with hydrogen concentrations ranging from 0 to 25%. An EQR of 1 was chosen for the methane–hydrogen–air mixture. The intake system consists of dual pipelines with the intake velocity of the methane–hydrogen mixture set at 1 m s^–1^ to maintain an EQR of 1. Correspondingly, adjustments were made to the intake velocity of the air pipeline, as indicated in Table 3. Figure 3 provides a schematic diagram illustrating the cross-sectional EQR distribution within the combustion chamber.

**Table 3:** Intake Velocity of Air Tube under Different Hydrogen Mixing Ratios

hydrogen mixing ratio (%)	methane–hydrogen gas (m·s^–1^)	air (m·s^–1^)	EQR
0	1	8.5837	1
5	1	8.5418	1
10	1	8.4949	1
15	1	8.4444	1
20	1	8.3906	1
25	1	8.3262	1

The external ambient temperature and the initial temperature of the premixed gas are both set to 300 K. The nonadiabatic walls of the combustion chamber experience convective heat transfer with a convective heat transfer coefficient of 25 W·(m^2^·K)^−1^

The governing equations are discretized and solved, and to ensure convergence of the calculations, a variable time step algorithm is employed. The initial time step is set to 1 × 10^–7^, the minimum time step is set to 1 × 10^–8^, and the maximum time step is set to 1 × 10^–5^.

Addressing the initialization issue of the flow field, a steady-state solver is initially utilized, running for 7000 steps to ensure uniformity and filling of the premixed gas within the combustion chamber. Subsequently, a transient solver is employed to simulate combustion.

The fundamental principle for selecting porous media materials is to choose materials with good heat transfer performance, strong resistance to thermal shocks, high heat resistance, and a certain level of mechanical strength. In this study, a high-temperature resistant nonmetallic porous media material, Al_2_O_3_, is chosen as the porous media material.^26^ The parameters of the combustion chamber and porous media required for the calculations can be found in Table 4.

**Table 4:** Comchamber and Porous Media

parameter		numerical value	unit
wall face convection heat transfer coefficient		25	W·(m^2^·K)^−1^
aperture	mixed area	2.9 × 10^–4^	m
	combustion zone	1.52 × 10^–4^	
porosity	mixed area	0.4	
	combustion zone	0.87	
thermal conductivity		6	W·(m·K)^−1^
emissivity		0.28	
absorption coefficient		3.7	m^–1^
scattering coefficient		28	m^–1^
density		3987	kg·m^–3^

## 3 Flame Temperature Measurement Experiment

### 3.1 Experimental Device and Burner Structure

**Figure 4:**
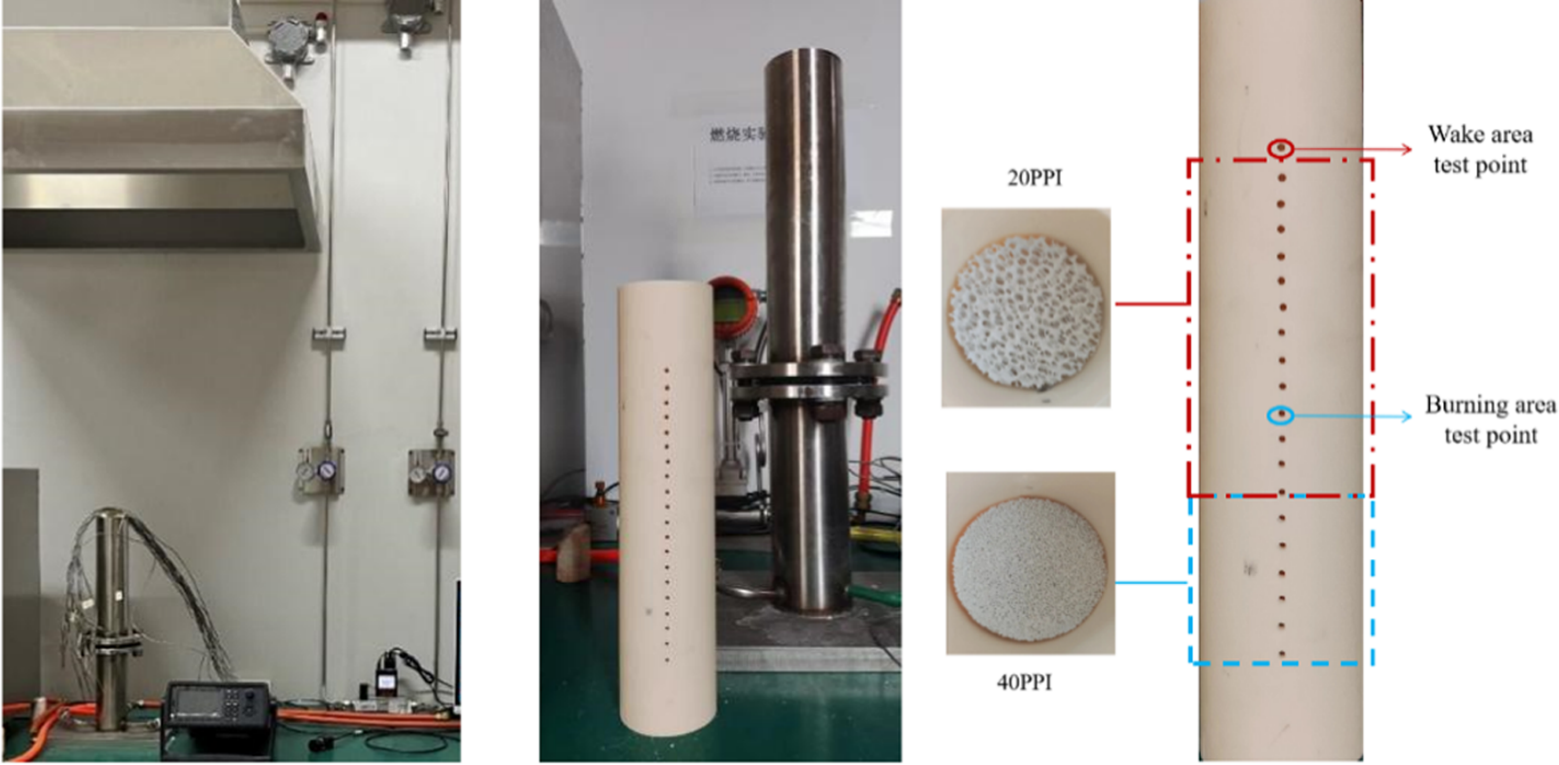
Experimental facility.

The experimental setup and burner configuration are illustrated in Figure 4. The setup consists of a porous burner, a fuel–air supply system, and a data acquisition system. Methane, hydrogen, and airflow rates are controlled by three mass flow controllers to meet the desired conditions at a fixed EQR of 1. To maintain stable pressure, laboratory air is supplied by a compressor connected to a storage tank and then filtered and dried through a dryer before entering the experimental apparatus. The primary equipment for data collection in this experiment includes thermocouples, a data acquisition device, and a computer. The burner itself is a ceramic tube with an inner diameter of 50 mm, a length of 260 mm, and a wall thickness of 5 mm. On one side of the tube wall, 20 evenly distributed test holes with a diameter of 2 mm are present for temperature measurement (identified as test points in the figure). An 80 mm long alumina porous media with a porosity of 20 PPI was positioned upstream for combustion testing, while a 40 mm long alumina porous media with a porosity of 40 PPI was positioned downstream to prevent flashback and promote gas premixing

### 3.2 Effect of Hydrogen Ratio on Flame Temperature

This section primarily focuses on the model validation of the PMB. The temperature measurements in the simulated flame were taken at the central temperature point of the cross section at a distance of 130 mm within the PMB. The thermocouple was employed to measure the temperature of the flame in the experiment. The thermocouples were inserted into the combustion zone of the PMB with a porosity of 20 PPI, corresponding to the simulated experimental test point. The data acquisition system was configured to collect data at intervals of 0.5 s, and readings were taken when the flame temperature reached a steady state.

**Figure 5:**
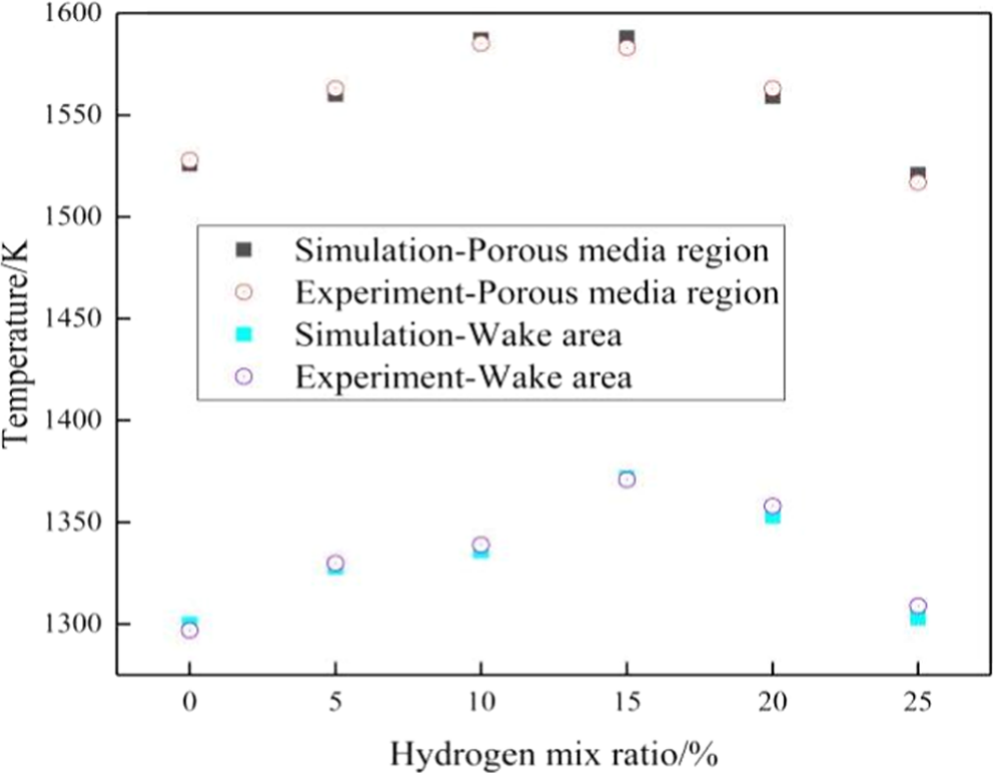
Relationship between the flame temperature and hydrogen content ratio.

For visual analysis, Figure 5 presents a line graph illustrating the relationship between the flame temperature and hydrogen blending ratio. The data indicates that the temperature in the porous media region is generally higher by 220 K compared to the downstream region. The increase in hydrogen blending ratio has a significant impact on the combustion flame temperature. From 0 to 15% hydrogen blending ratio, the temperature increases with the increase in the blending ratio, reaching its peak at 15%. Subsequently, as the blending ratio reaches 20 to 25%, the temperature gradually decreases. The rising and falling trends of the temperature curves in both the experimental and simulation data are consistent, validating the feasibility of the simulation model.

## 4 Results and Discussion

**Figure 6:**
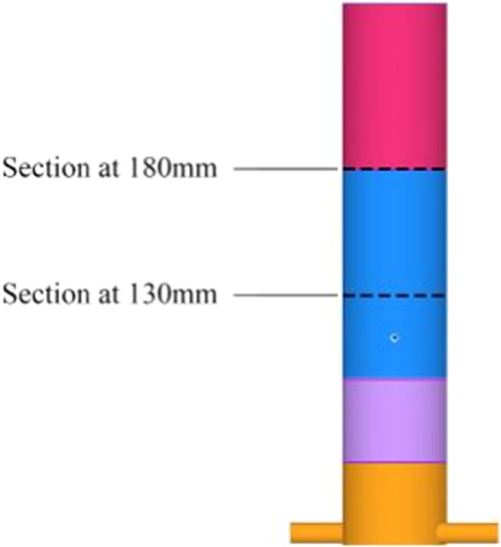
Schematic diagram of cross-sectional location.

Figure 6 shows the critical monitoring locations. In order to have a more complete observation of the temperature characteristics within the PMB, the location is chosen to be 130 mm from the bottom of the combustor. In order to monitor the emission characteristics of the combustion products more effectively, we chose the location to be 180 mm from the bottom of the combustor.

### 4.1 Combustion Characteristics

**Figure 7:**
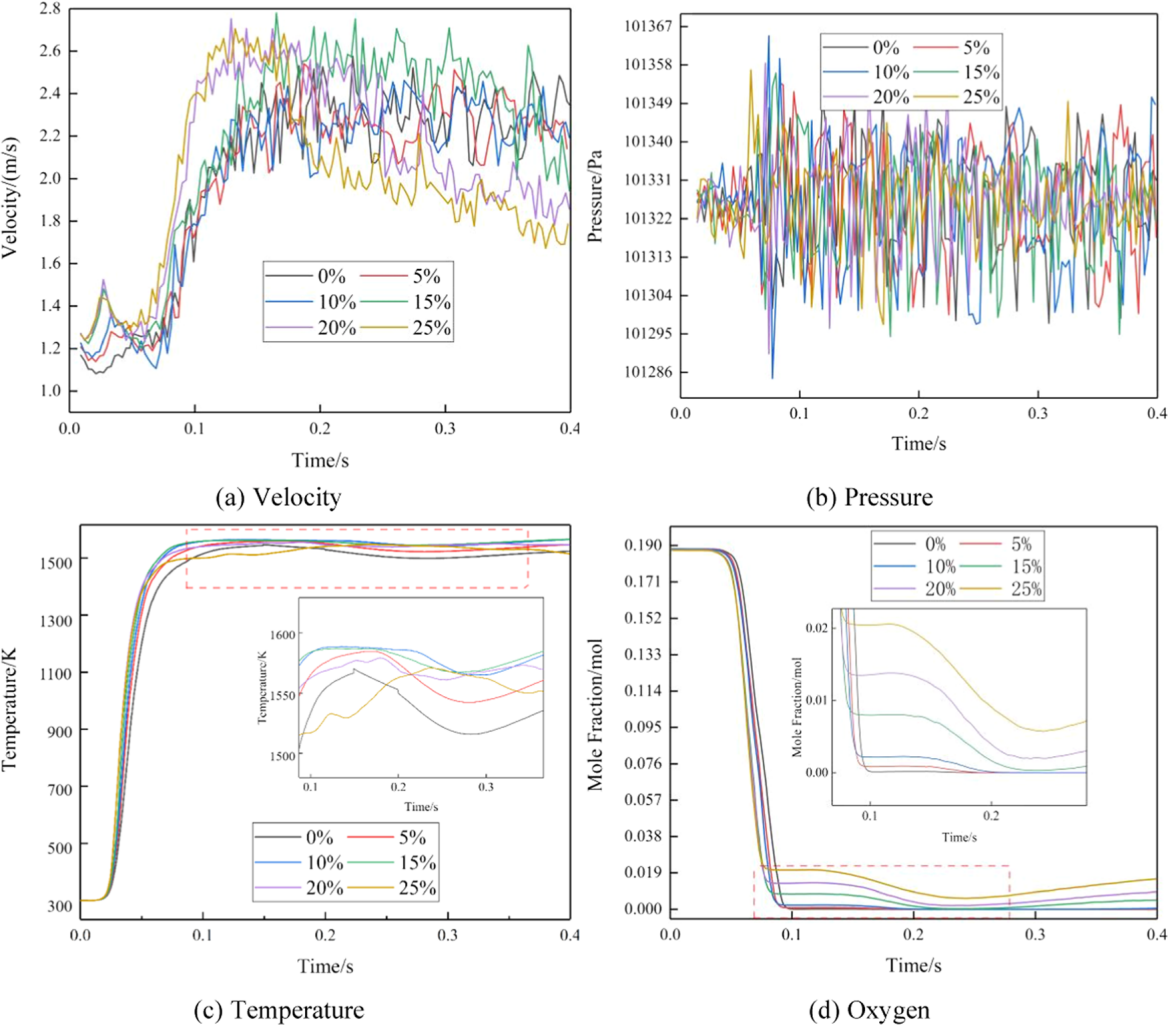
Physical property curve of the 130 mm burner.

This study specifically investigates the combustion characteristics within the porous media, focusing primarily on the data from the porous media region. Figure 7 illustrates the temporal variations of the average temperature, oxygen molar fraction, velocity, and pressure at the cross-sectional location of 130 mm within the central area of the “Burning area” in the combustor.

This cross section is positioned at the center of the high-temperature region within the porous media combustion zone, making it an optimal location for studying its combustion characteristics (as indicated in Figure 6).

In Figure 7, multiple peaks are observed in the pressure (*a*) and velocity (*b*) trends, signifying the occurrence of resonance combustion. Resonance combustion arises from the accumulation and amplification of small vibrations. In this study, during the combustion process in the combustion chamber, the uneven distribution of pores within the porous media causes slight variations in the pressure at the combustion surface. Consequently, the fuel burning rate also undergoes corresponding changes over time. Simultaneously, as pressure waves propagate through the gas and reflect off the walls of the combustion chamber, the intensity of the pressure waves gradually weakens due to the round-trip propagation and reflection processes. However, when the pressure wave coincides with the variation in the fuel burning rate upon returning to the combustion chamber surface, the pressure wave gradually strengthens, leading to resonance. This results in an increased burning rate and a drastic rise in chamber pressure, leading to the appearance of pressure peaks and the occurrence of resonance combustion.^27−29^

Under the influence of resonance combustion, pressure values exhibit instability with the highest pressure difference reaching 60 Pa. Velocity variations tend toward a stable state for hydrogen blending ratios ranging from 0 to 15%. However, for blending ratios of 20 to 25%, velocity values experience significant fluctuations due to the instability of hydrogen combustion and large pressure oscillations. The fluctuation in heat release is the underlying cause of these oscillations, with the main factors affecting unstable heat release being the flame surface area and fuel EQR fluctuation. These factors influence heat release, leading to the two aforementioned outcomes and ultimately resulting in resonance combustion. One intuitive reason for this is the tendency of fuel species, such as methane and hydrogen, to undergo flame lifting during combustion.

Temperature trends are depicted in Figure 7(c), where all curves except the 25% curve exhibit a temperature dip between 0.20 and 0.30 s. Figure 7(d) illustrates the variation in the oxygen concentration. The addition of hydrogen increases the combustion rate and accelerates the consumption of oxygen. As the hydrogen blending ratio in the fuel increases, the oxygen concentration reaches equilibrium first for high blending ratios. However, during the time interval of 0.20 to 0.30 s, a trough in the oxygen concentration is observed. This phenomenon arises due to the superposition of pressure waves generated by the excitation source within the 0.20 to 0.30 s time frame, resulting in resonance combustion and a sudden increase in the combustion rate. As a consequence, the oxygen supply becomes insufficient, thereby affecting the combustion process.

Temperature profiles of the four curves with hydrogen blending ratios ranging from 0 to 15% exhibit a gradual increase, while the two curves with blending ratios of 20 to 25% display a gradual decrease. At a combustion time of 0.40s, when the combustion temperature stabilizes, temperatures for the blending ratios of 0 to 15% are 1528, 1563, 1585, and 1583 K, respectively. For the blending ratios of 20 to 25%, temperatures are 1569 and 1517 K, with a temperature difference of 68K between the highest and lowest values. Compared to CH_4_, H_2_ has a lower activation energy when participating in the reaction, making the reaction between H_2_ and O_2_ more favorable and resulting in a higher combustion rate. Due to the fewer intermediate reaction steps involved in the H_2_–O_2_ reaction compared to the CH_4_–O_2_ reaction,^30^ which has more complex intermediates, H_2_ blending ratios of 0 to 15% exhibit a gradual increase in temperature. However, the heating value of CH_4_ (39.83 MJ m^–3^) is higher than that of H_2_ (12.70 MJ m^–3^). As the hydrogen content increases, the proportion of CH_4_ decreases in the premixed gas, leading to a decrease in the heat released during combustion and subsequently causing a gradual temperature decrease. Hence, the temperature decreases for blending ratios of 20 to 25%.

**Figure 8:**
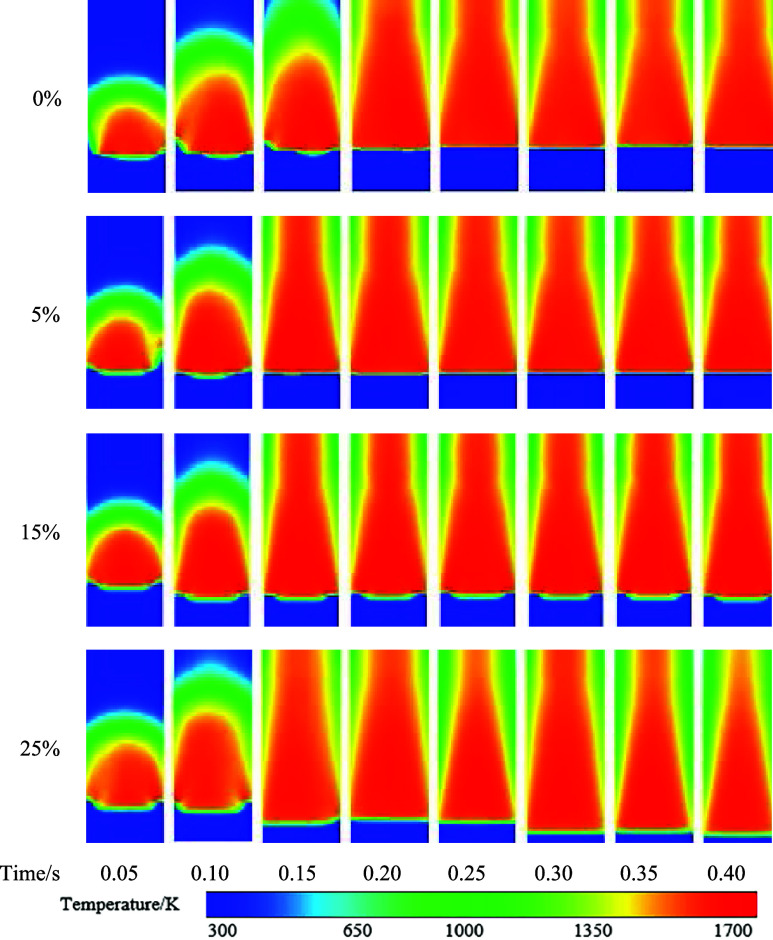
Temperature distribution.

The distribution of temperature in the vertical cross section at the center of the combustion zone at different times is shown in Figure 8. Due to the characteristics of hydrogen gas, the occurrence of reignition becomes more prominent with an increase in hydrogen concentration. Notably, significant reignition phenomena start to appear at a hydrogen concentration of 15%. When the hydrogen concentration reaches 15 and 25%, the high-temperature burning area in the porous media shifts downward due to reignition, but it is hindered by the mixed-zone region in the porous media.

The high-temperature locations predominantly concentrate within the porous media region. The peak temperature of flame combustion exhibits an initial increase in the temperature of the porous media region from 5 to 15%, accompanied by a slight widening of the high-temperature region. However, beyond 15 up to 25% hydrogen concentration, the temperature decreases, resulting in a slight reduction in the width of the high-temperature region. The high-temperature region in the combustion chamber primarily concentrates in the porous media combustion section, while the wake area lacks the presence of a porous media, leading to a narrower high-temperature region compared with the porous media section in the combustion zone. Due to the unique pore structure of the porous media, it is more conducive to enhanced gas flow and mixing in the combustion zone. This augmented mixing effect results in the expansion of the flame over a wider area, thereby increasing the flame width. The utilization of porous media offers a larger surface area and greater pore space, which, in turn, provides additional reactive surfaces and pathways for fuel diffusion. Consequently, this amplified diffusion path promotes the increased participation of the fuel in the combustion process and leads to a wider flame.

### 4.2 Carbon Emission

**Figure 9:**
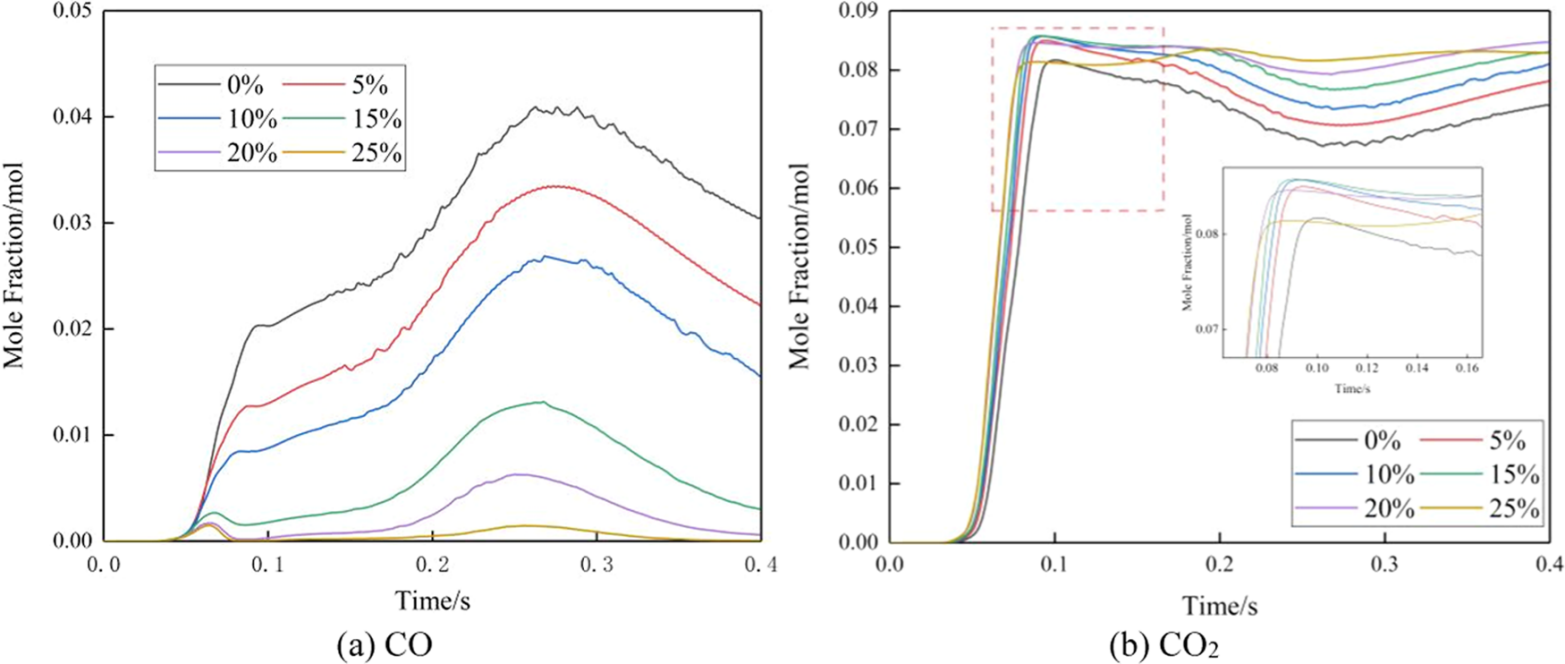
Physical property curve of the 180 mm burner.

Figure 9 illustrates the temporal variation of species molar concentrations at the 180 mm cross section of the burner. At 0.05 s, the flame reaches the 180 mm position of the burner, leading to a significant decrease in the O_2_ concentration due to combustion chemical reactions. Concurrently, carbon monoxide and carbon dioxide are generated as a result of the combustion reactions.

The plots of oxygen, carbon monoxide, and carbon dioxide reveal a distinct region of increased combustion rate between 0.20 and 0.30 s. The O_2_ and CO_2_ curves exhibit a trough around 0.20 to 0.30 s, while the CO curve shows a peak during the same time frame. This phenomenon can be attributed to the influence of resonant combustion, which leads to an accelerated combustion rate. The filling rate of combustion gases fails to keep up with the rate of combustion consumption, resulting in a lower O_2_ content during this period compared to the average oxygen content after stable combustion. Factors such as the high temperature and oxygen concentration also impact the generation of CO. The decrease in oxygen content promotes the formation of CO. As the amount of carbon entering the combustion chamber remains constant during the same time interval, the increase in CO leads to a decrease in CO_2_.

As the amount of H_2_ injection increases and CH_4_ decreases, the overall carbon emissions are reduced. Comparing the molar variations of carbon dioxide and carbon monoxide, it can be observed that the emission ratio of CO to CO_2_ decreases with an increase in hydrogen blending ratio (as shown in Table 5). The emission of CO decreases proportionally.

**Table 5:** CO/CO_2_ Values at Different Hydrogen-Doped Ratios

hydrogen mixing ratio	0%	5%	10%	15%	20%	25%
CO/CO_2_	0.6113	0.4712	0.3584	0.1613	0.0712	0.0161

**Figure 10:**
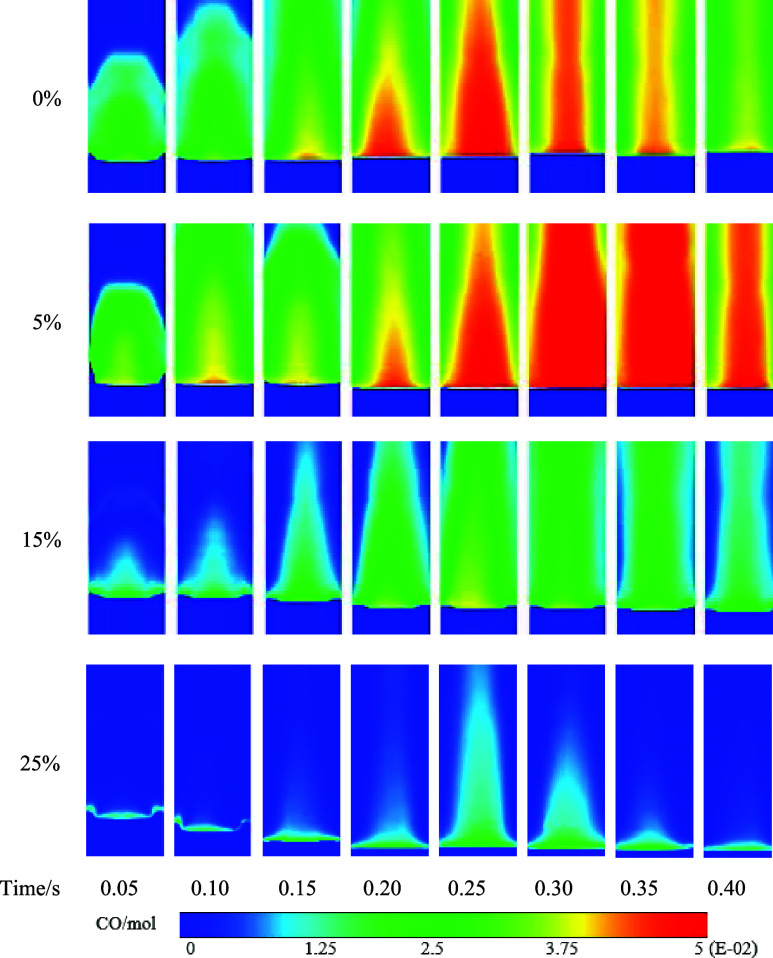
Carbon monoxide distribution cloud map.

Figure 10 illustrates the distribution of CO at different time points in the burning area. By comparison of the evolution of CO emissions over time, it is observed that CO is primarily generated in the central region of the combustion section, and its production decreases with an increase in hydrogen concentration. This reduction in CO generation can be attributed to the promoting effect of high temperatures on the formation of CO formation. Consequently, the region of CO generation follows the downward shift of the high-temperature zone within the combustion chamber, resulting in the concentration of carbon monoxide in the high-temperature region generated by the porous media combustion section.

By comparing the temporal variations of CO emissions, it is observed that the peak CO emission for pure methane combustion occurs between 0.30 and 0.35 s, while the peak CO emission for the hydrogen-enriched mixture occurs between 0.25 and 0.30 s. The results show that hydrogen exhibits higher reactivity compared to that of methane. When hydrogen is induced into the combustion process, it enhances the overall combustion rate and promotes faster oxidation reactions. It also leads to an earlier release of carbon monoxide as an intermediate product. The presence of hydrogen in the fuel mixture alters the flame characteristics, with hydrogen having a higher flame speed and a shorter residence time within the combustion zone. Consequently, the time available for the conversion of CO to CO_2_ is reduced, resulting in an earlier peak in CO emissions.

### 4.3 Nitrogen Emissions

**Figure 11:**
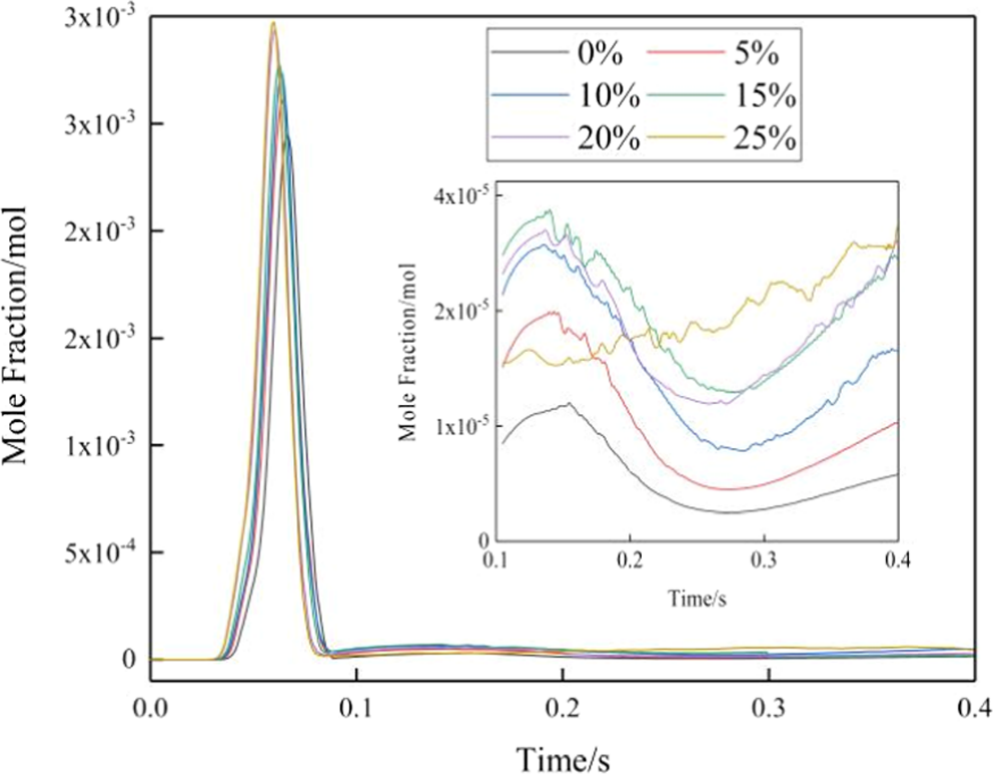
NOx emission.

Figure 11 illustrates the variation of NOx emissions at the cross section of the burner with a diameter of 180 mm. In porous media combustion, the instantaneous NOx emissions reach a peak between 0s and 0.1 s due to excessively high ignition temperatures. The emissions then reach their maximum at 0.05s. As the hydrogen concentration increases, the NOx emissions increase in both magnitude and rate, reaching their peak faster. The NOx emissions exhibit a gradual increase in the range of 0–15%, but a decrease is observed in the range of 20–25%. This decline can be attributed to the strong dependence of NOx emissions on the temperature. It is evident from the curve observations that the changes in the NOx emissions correspond to the changes in the temperature. The concentration of NOx initially rises and then decreases, but the total emissions first decrease and then increase.

Figure 11 reveals the presence of a trough between 0.20 and 0.30 s after the stabilization of NOx emissions. During the combustion process of methane, nitrogen oxides, mainly nitric oxide and nitrogen dioxide, are generated. The production and emissions of these nitrogen oxides are closely related to the combustion mode, particularly the combustion temperature and air-fuel ratio. Factors influencing the formation of nitrogen oxides include temperature, oxygen content, reaction time, and chemical characteristics. Sufficient oxygen supply promotes the conversion of nitrogen in the fuel to NOx. However, between 0.20 and 0.30 s, the insufficient supply of oxygen, possibly due to resonant combustion and other reasons, inhibits the production of nitrogen oxides.

**Figure 12:**
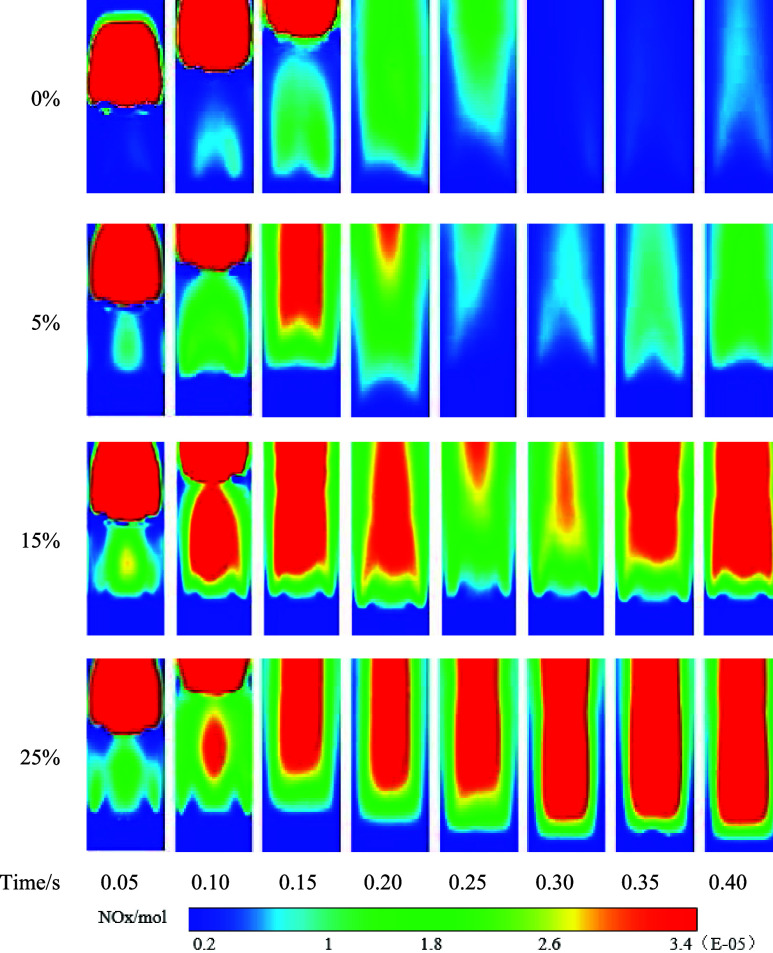
NOx distribution cloud map.

Figure 12 depicts the distribution of NOx in the entire burning area at different time points. The generation of NOx primarily concentrates near the central axis. As the high-temperature zone shifts downward, the generation of NOx follows suit, as it is directly influenced by the temperature. This correlation between NOx generation and temperature explains the movement of NOx along with the high-temperature region.

In porous media combustion, the generation of NOx increases with an increase in hydrogen concentration in the premixed gas. This phenomenon occurs primarily due to the higher flame temperature of hydrogen gas compared to methane, reaching nearly 300 °C higher. Additionally, the ignition delay time of hydrogen is more than 3 times lower than that of natural gas. When the hydrogen concentration in the fuel is higher, the fuel reactivity changes, leading to issues such as excessive combustion time and increased nitrogen oxide emissions. This situation can potentially result in overheating of the combustion chamber, leading to a gradual increase in the molar fraction of NOx.

It can be clearly demonstrated that with an increase in the blending ratio of hydrogen, the instantaneous NOx reaches the cross section at 180 mm more quickly. It may be due to the higher chemical reactivity of hydrogen. With its faster reaction rate, hydrogen leads to a shorter residence time within the combustion zone, thereby reducing the reaction time for nitrogen species. As a result, the opportunity to convert nitrogen to less active substances of molecular nitrogen is reduced. This favors the formation of nitrogen oxides since nitrogen-containing radicals and intermediate species have a higher likelihood of reacting with oxygen to generate nitrogen oxides. Therefore, the instant production of NOx tends to increase with the addition of hydrogen.

## 5 Conclusions

In this study, we developed a geometric model of a cylindrical double-layer porous burner to investigate the transient combustion flame characteristics of methane–hydrogen blending within porous media. Our focus was on understanding the impact of hydrogen blending ratios on combustion parameters and providing insights for optimizing PMB. As the hydrogen blending ratio increases, we observed an accelerated diffusion of the flame within the porous combustion zone, leading to an enhanced combustion rate and a subtle adjustment in flame width proportional to the hydrogen content. The peak flame temperature exhibited an initial increase of up to 15% hydrogen blending, followed by a decrease from 15 to 25%, with the maximum temperature achieved at a blending ratio of 15%. Notably, the high-temperature region is predominantly concentrated in the porous combustion section, emphasizing its dependence on the presence of porous media. Resonant combustion phenomena manifested between 0.20 and 0.30 s during the combustion process, resulting in intensified combustion rates and inadequate O_2_ supply. Within this interval, specific parameters, such as temperature, CO_2_, and NOx emissions, showed low peaks, while CO emissions exhibited a prominently high peak. Moreover, an increase in the hydrogen blending ratio correlated with reduced total carbon emissions and a decreasing trend in the CO/CO_2_ values. Significantly, instantaneous NOx generation peaked between 0.02 and 0.10 s, and during the stable emission period of 0.10 to 0.40 s, NOx concentrations initially rose and then declined, while total emissions exhibited a nonmonotonic pattern. These findings elucidate the nuanced transient combustion flame characteristics associated with methane–hydrogen blending within porous media, providing valuable insights for optimizing the design and performance of PMB.
